# Variability of Spectral Tristimulus Values

**DOI:** 10.6028/jres.065A.050

**Published:** 1961-12-01

**Authors:** Isadore Nimeroff, Joan R. Rosenblatt, Mary C. Dannemiller

## Abstract

As the spectral tristimulus values of the CIE Standard Observer System for Colorimetry are measurable quantities, their variabilities should be known. This paper describes a procedure for deriving “within” and “between” variances and covariances in the spectral tristimulus values, based on color-matching data for individual observers. The “within” variances are based on the replications of color-mixture data by an observer. The “between” variances are based on differences among the color-mixture data of individual observers. A statistical model is given for the system in which the experimental data are obtained. Formulas for expected values (means), variances, and covariances are developed. Variances and covariances belonging to different sources of uncertainties in the experimental data are considered. A procedure is developed for determining the uncertainties in the constants of a linear transformation to a system analogous to the present CIE system. The formulas for variances and covariances after linear transformation are given, for a rigorous empirically-based choice, and also for an arbitrary choice of transformation constants. The complete standard observer system for every 10 m*μ* consisting of means, variances, and covariances derived from an arbitrary transformation, is listed. The between-observer variabilities are found to be about 10 percent of the averages of the color-mixture data and the average ratio of the between-observer variabilities to the within-observer variabilities is found to be about 5.7.

## 1. Introduction

Since 1931 the International Commission on Illumination has recommended the use of a Standard Observer System for Colorimetry [1].^1^ This system defines the manner in which spectral data for materials are to be reduced to three numbers, called tristimulus values, that describe colors of emitted, reflected, or transmitted lights. The defining equations for these tristimulus values are:

$$\begin{array}{l} X\equiv \int_{0}^{\infty }{\bar{x}}_{\lambda }N_{\lambda }T_{\lambda }d\lambda ≐\sum_{0}^{\infty }{\bar{x}}_{\lambda }N_{\lambda }T_{\lambda }\Delta_{\lambda }\\ Y\equiv \int_{0}^{\infty }{\bar{y}}_{\lambda }N_{\lambda }T_{\lambda }d\lambda ≐\sum_{0}^{\infty }{\bar{y}}_{\lambda }N_{\lambda }T_{\lambda }\Delta_{\lambda }\\ Z\equiv \int_{0}^{\infty }{\bar{z}}_{\lambda }N_{\lambda }T_{\lambda }d\lambda ≐\sum_{0}^{\infty }{\bar{z}}_{\lambda }N_{\lambda }T_{\lambda }\Delta_{\lambda } \end{array}$$

The quantities ${\bar{x}}_{\lambda }$, ${\bar{y}}_{\lambda }$ and ${\bar{z}}_{\lambda }$ are called spectral tristimulus values and are intended to be descriptive of the spectral-light response of the average human observer with normal color vision. The quantity *N*_λ_ describes the spectral emittance of light sources and the quantity *T*_λ_ describes the spectral character of the reflecting or transmitting materials.

Tristimulus values are usually reduced to chromaticity coordinates by the equations:

x=X/S,y=Y/S,andz=Z/S,

where *S* is the sum of the tristimulus values *X, Y*, and *Z*. As ${\bar{x}}_{\lambda }$, ${\bar{y}}_{\lambda }$ and ${\bar{z}}_{\lambda }$, *N*_λ_, and *T*_λ_ are measured quantitles, they are subject to measurement uncertainty. Nimeroff [2,3] has treated, by means of propagation of error theory, the manner in which variabilities in *T*_λ_ and in *N*_λ_ affect the chromaticity coordinates, *x, y*, and *z*.

The general problem and several special cases of propagation of errors in tristimulus colorimetry have been treated by Nimeroff [3]. In that treatment the mean spectral tristimulus values, $\bar{x}$, $\bar{y}$ and $\bar{z}$, were estimated by averaging the mean CIE (17 observers) and mean Stiles’ 2°- and 10°-field pilot data (10 observers each). The variances in these values were estimated in the usual manner by using deviations of these three mean data from the estimated overall mean values; the covariances were ignored. The variances as well as the covariances should, however, be more fundamentally estimated; that is, they should be estimated from differences among color-mixture functions of individual observers. Such data became available in 1959. This paper describes how this fundamental estimation of the between-observer variances and covariances may be made for the 10°-field color-mixture data of the 53 observers of Stiles-Burch [4] and the 27 observers of Speranskaya [5], and gives estimates of the average within-observer variances and covariances of two observers, one with 4 and the other with 5 replications. The estimates of covariances are developed on the basis of the data of the 53 observers of Stiles-Burch.

## 2. Statistical Model

Fundamental color-matching data are obtained on a device where an observer is presented two fields which he is asked to color-match, by adjusting the amounts of three primary colors. In one field, there is a fixed amount, *E*_λ_, of a given spectral color of wavelength λ. The three adjustable primaries having wavelengths 645.2, 526.3, and 444.4 m*μ* may be denoted *R, G, B*, respectively. One is added to the field containing the given color, and the other two are mixed in the second field. If *R*_λ_ is the amount of the Red (*R*) primary (in energy units) used in matching *E*_λ_, and *G*_λ_ and *B*_λ_ are similarly defined, then the condition of color-matching may be expressed:

$$E_{\lambda }\overset{m}{\equiv }R_{\lambda }+G_{\lambda }+B_{\lambda },$$ (1)

where the primary which was mixed with the given color is represented by a negative quantity on the right-hand side of this symbolic equation.

The complete set of color-mixture data for a single observer consists of the amounts of the three primaries used in matching a series of spectrum colors sampling the entire visible portion. These data are adjusted so that, in suitable units, (*R*_λ_, *G*_λ_, *B*_λ_) represent the amounts used in matching a unit amount (energy) of the spectrum color at wavelength λ.

The empirical results for the *i*th observer may be denoted (*R*_λ_*_i_*, *G*_λ_*_i_*, *B*_λ_*_i_*). Omitting the subscript λ we consider the following model:

$$\begin{array}{l} R_{i}=R+b_{Ri}+\epsilon_{Ri}\\ G_{i}=G+b_{Gi}+\epsilon_{Gi}\\ B_{i}=B+b_{Bi}+\epsilon_{Bi}, \end{array}$$ (2)

where each of *R_i_*, *G_i_*, *B_i_* is represented as the sum of three terms: *R*, the average amount which would be used in color-matching in a population of observers with normal color vision; the “bias” *b_Ri_* of the *i*th observer; and an “error” *ϵ_Ri_.* The bias *b_Ri_* is the amount by which the *i*th observer’s “true” average differs from the population average *R.* The error *ϵ_Ri_* is the difference between a given observation *R_i_* and the *i*th observer’s true average which is (*R+b_Ri_*). The terms of the second and third eqs of (2) are similarly defined. The expected value (true average) of error *ϵ_i_* is assumed to be zero; thus, for the *i*th observer,

$$\begin{array}{l} E_{i}\left[R_{i}\right]=R+b_{Ri}\\ E_{i}\left[G_{i}\right]=G+b_{Gi}\\ E_{i}\left[B_{i}\right]=B+b_{Bi} \end{array}$$

where *E_i_* denotes the operation of averaging (taking the expected value) over the hypothetical set of repeated observations by the *i*th observer.

The “within” variance for the *i*th observer (for the red primary, say) is the average squared deviation of *R_i_* from *E_i_*[*R_i_*],

$$V_{i}\left(R_{i}\right)=E_{i}{\left[R_{i}-E_{i}\left[R_{i}\right]\right]}^{2}=E_{i}\left[{\epsilon^{2}}_{Ri}\right].$$

If all observers have the same “within” variance, then this common variance is denoted by ${\;}_{w}\sigma_{R}^{2}$; when it is desired to specify the dependence of this variance on the wavelength of the spectrum color being matched, the subscript λ is restored and the within variance is denoted by ${\;}_{w}\sigma_{R\lambda }^{2}$.

Now, referring again to eq(2), the average or expected value of *R_i_* over the whole population of observers is *R*; *E*[*R_i_*] *= R*, where *E* (without subscript *i*) denotes the operation of averaging over the population of observers. Therefore, it follows that *E*[*b_Ri_*] = 0.

The “between” variance, in the population of observers, is the average squared deviation of the *i*th observer’s average (*R+b_Ri_*) from the population average *R*,

$${\;}_{b}\sigma_{R}^{2}=E\left[b_{Ri}^{2}\right].$$

It is assumed that the error *ϵ_Ri_* is statistically independent of the observer bias *b_Ri_.* Thus, the total variance $\sigma_{R}^{2}$ of the observed value *R_i_* for one observation by one observer is the sum of the within and between variances, and similarly for the green and blue primaries:

$$\begin{array}{l} \sigma_{R}^{2}={\;}_{b}\sigma_{R}^{2}+{\;}_{w}\sigma_{R}^{2}\\ \sigma_{G}^{2}={\;}_{b}\sigma_{G}^{2}+{\;}_{w}\sigma_{G}^{2}\\ \sigma_{B}^{2}={\;}_{b}\sigma_{B}^{2}+{\;}_{w}\sigma_{B}^{2}. \end{array}$$ (3)

Although it is perhaps reasonable to assume that an observer’s “errors” are not correlated with his “biases”, the analyses of empirical data below will show that it cannot be assumed that the three observations *R_i_, G_i_, B_i_* for the *i*th observer are independent. Accordingly, it is necessary to know the covariances. The “between” covariances are:

$$E\left[b_{Ri}b_{Gi}\right],\;E\left[b_{Ri}b_{Bi}\right],\;E\left[b_{Gi}b_{Bi}\right].$$

The “within” covariances are:

$$C_{i}\left(R_{i},G_{i}\right)=E_{i}\left[\left(R_{i}-E_{i}\left[R_{i}\right]\right)\;\left(G_{i}-E_{i}\left[G_{i}\right]\right)\right]=E_{i}\left[\epsilon_{Ri}\epsilon_{Gi}\right].$$

When we assume that the within covariances are equal for all observers, we may then write the total covariances as:

$$\begin{array}{l} C\left(R,G\right)={\;}_{b}\sigma_{RG}+{\;}_{w}\sigma_{RG},\\ C\left(R,B\right)={\;}_{b}\sigma_{RB}+{\;}_{w}\sigma_{RB},\\ C\left(G,B\right)={\;}_{b}\sigma_{GB}+{\;}_{w}\sigma_{GB}, \end{array}$$ (4)

where, for example,

$${\;}_{b}\sigma_{RG}=E\left[b_{Ri}b_{Gi}\right]$$

and

$${\;}_{w}\sigma_{RG}=E_{i}\left[\epsilon_{Ri}\epsilon_{Gi}\right].$$

No attempt will be made in this paper to estimate the within and between covariances separately; estimates are given for the total correlations such as *ρ_RG_*, defined by eqs (3), (4), and

$$C\left(R,G\right)=\rho_{RG}\sigma_{R}\sigma_{G}$$ (5)

The total variances and covariances are estimated in the usual way. For example, the estimate for $\sigma_{R}^{2}$ is

$${\hat{\sigma }}_{R}^{2}=\sum_{i=1}^{n}{\left(R_{i}-\bar{R}\right)}^{2}/\left(n-1\right)$$

where *n* is the number of observers and $\bar{R}$ is the average of the *R_i_* for the *n* observers.

## 3. Rigorous Transformation to a System Analogous to CIE

Spectral tristimulus values, $\bar{r}$, $\bar{g}$, $\bar{b}$, have been derived from measured color-matching data of the primaries, wavelengths 645.2, 526.3, and 444.4 m*μ.* An empirically-based system of spectral tristimulus values, $\bar{x}$, $\bar{y}$, $\bar{z}$, analogous to the present CIE system, may be derived from these tristimulus values, $\bar{r}$, $\bar{g}$, $\bar{b}$, by means of the eqs (6) below. If we wish to locate the chromatidty coordinates of the equal-energy point (*x_n_, y_n_, z_n_*) at the point (1/3, 1/3, 1/3) we must set a restriction that $\Sigma {\bar{x}}_{\lambda }=\Sigma {\bar{y}}_{\lambda }=\Sigma {\bar{z}}_{\lambda }$. This is the purpose of the adjustment factors, *f_x_*, *f_y_* and *f_z_*, respectively.

$$\begin{array}{l} {\bar{x}}_{\lambda }=f_{x}\left(k_{1}{\bar{r}}_{\lambda }+k_{2}{\bar{g}}_{\lambda }+k_{3}b_{\lambda }\right)=f_{x}{\bar{x}}_{\lambda }^{\prime }\\ {\bar{y}}_{\lambda }=f_{y}\left(k_{4}{\bar{r}}_{\lambda }+k_{5}{\bar{g}}_{\lambda }+k_{6}{\bar{b}}_{\lambda }\right)=f_{y}{\bar{y}}_{\lambda }^{\prime }\\ {\bar{z}}_{\lambda }=f_{z}\left(k_{7}{\bar{r}}_{\lambda }+k_{8}{\bar{g}}_{\lambda }+k_{9}{\bar{b}}_{\lambda }\right)=f_{z}{\bar{z}}_{\lambda }^{\prime }. \end{array}$$ (6)

We may impose two additional restrictions on the system:

1. That the tristimulus values be not less than zero.
2. That no one of the functions $\bar{x}$, $\bar{y}$, $\bar{z}$, be equivalent to a linear combination of the other two. This restriction is established if the determinant of coefficients *k_i_* does not vanish, that is,

$$\left|\begin{array}{ccc} k_{1} & k_{2} & k_{3}\\ k_{4} & k_{5} & k_{6}\\ k_{7} & k_{8} & k_{9} \end{array}\right|\neq 0.$$ (6a)

If all the luminosity, *V*_λ_ is placed in the ${\bar{y}}_{\lambda }$ primary, and if *f_y_* is set equal to 1, then *k*_4_, *k*_5_, and *k*_6_ are determined by

$$V_{\lambda }={\bar{y}}_{\lambda }^{\prime }=L_{r}{\bar{r}}_{\lambda }+L_{g}{\bar{g}}_{\lambda }+L_{b}{\bar{b}}_{\lambda }=k_{4}{\bar{r}}_{\lambda }+k_{5}{\bar{g}}_{\lambda }+k_{6}{\bar{b}}_{\lambda }$$ (7)

thereby making *k*_4_*=L_r_*, *k*_5_*=L_g_* and *k*_6_=*L_b_*. The symbols *L_r_, L*_g_, and *L_b_* are the luminous units of th*e* $\bar{r}\;\bar{g}\;\bar{b}$ system.

The solutions for the *k_i_*’s in the $\bar{x}$ and $\bar{z}$ spectral tristimulus functions are not obtained as directly as those for the *k_i_*’s in the $\bar{y}$ function. Equations (8) to (8e) below show how a solution of the *k_i_*’s in the $\bar{x}$ function may be obtained.

The $\bar{x}\prime$ equation (eq 8) contains three *k_i_’*s to be determined. A unique solution for *k*_1_, *k*_2_, and *k*_3_ requires three simultaneous equations. By assigning, tentatively, a value of unity to *k*_1_ (eq 8a), no loss of generality is encountered that cannot be accounted for by the adjustment factor, *f_x_*, in eq (6).

$${\bar{x}}_{\lambda }^{\prime }=k_{1}{\bar{r}}_{\lambda }+k_{2}{\bar{g}}_{\lambda }+k_{3}{\bar{b}}_{\lambda }$$ (8)

$${\bar{x}}_{\lambda }^{\prime }={\bar{r}}_{\lambda }+k_{2}{\bar{g}}_{\lambda }+k_{3}{\bar{b}}_{\lambda }.$$ (8a)

When this is done, only two simultaneous equations are required to solve for the remaining two constants. The equations are set up by selecting, at two wavelengths (λ_1_ and λ_2_), numerical values for say, ${\bar{x}}_{1}$ and ${\bar{x}}_{2}$, that will satisfy the requirement that all the tristimulus values be positive. Equation (8a) then becomes

$${\bar{x}}_{1}^{\prime }=r_{1}+k_{2}{\bar{g}}_{1}+k_{3}{\bar{b}}_{1}$$ (8b)

$${\bar{x}}_{2}^{\prime }={\bar{r}}_{2}+k_{2}{\bar{g}}_{2}+k_{3}{\bar{b}}_{2}$$ (8c)

and the solutions for *k*_2_ and *k*_3_ are given by:

$$k_{2}=\left[\left({\bar{x}}_{1}^{\prime }-{\bar{r}}_{1}\right){\bar{b}}_{2}-\left({\bar{x}}_{2}^{\prime }-{\bar{r}}_{2}\right){\bar{b}}_{1}\right]/\left({\bar{g}}_{1}{\bar{b}}_{2}-{\bar{g}}_{2}{\bar{b}}_{1}\right)$$ (8d)

$$k_{3}=\left[\left({\bar{x}}_{2}^{\prime }-{\bar{r}}_{2}\right){\bar{g}}_{1}-\left({\bar{x}}_{1}^{\prime }-{\bar{r}}_{1}\right){\bar{g}}_{2}\right]/\left({\bar{g}}_{1}{\bar{b}}_{2}-{\bar{g}}_{2}{\bar{b}}_{1}\right).$$ (8e)

The constants *k_i_* of the $\bar{z}$ function are solved in a similar manner as shown by eqs (9) to (9e):

$${\bar{z}}_{\lambda }^{\prime }=k_{7}{\bar{r}}_{\lambda }+k_{8}{\bar{g}}_{\lambda }+k_{9}{\bar{b}}_{\lambda }$$ (9)

$${\bar{z}}_{\lambda }^{\prime }=k_{7}{\bar{r}}_{\lambda }+k_{8}{\bar{g}}_{\lambda }+{\bar{b}}_{\lambda }$$ (9a)

$${\bar{z}}_{3}^{\prime }=k_{7}{\bar{r}}_{3}+k_{8}{\bar{g}}_{3}+{\bar{b}}_{3}$$ (9b)

$${\bar{z}}_{4}^{\prime }=k_{7}{\bar{r}}_{4}+k_{8}{\bar{g}}_{4}+{\bar{b}}_{4}$$ (9c)

$$k_{7}=\left[\left({\bar{z}}_{3}^{\prime }-{\bar{b}}_{3}\right){\bar{g}}_{4}-\left({\bar{z}}_{4}^{\prime }-{\bar{b}}_{4}\right){\bar{g}}_{3}\right]/\left({\bar{r}}_{3}{\bar{g}}_{4}-{\bar{r}}_{4}{\bar{g}}_{3}\right)$$ (9d)

$$k_{8}=\left[\left({\bar{z}}_{4}^{\prime }-{\bar{b}}_{4}\right){\bar{r}}_{3}-\left({\bar{z}}_{3}^{\prime }-{\bar{b}}_{3}\right){\bar{r}}_{4}\right]/\left({\bar{r}}_{3}{\bar{g}}_{4}-{\bar{r}}_{4}{\bar{g}}_{3}\right).$$ (9e)

## 4. Variances and Covariances in a Rigorous Transformation

In general, variances and covariances based on propagation of error theory are:

$$\sigma_{U}^{2}={\sum_{n=1}^{k}\left(\frac{\partial U}{\partial v_{n}}\right)}^{2}\sigma_{v_{n}}^{2^{-}}+\sum_{m\neq n}\left(\frac{\partial U}{\partial v_{n}}\right)\;\left(\frac{\partial U}{\partial v_{m}}\right)\sigma_{v_{m}v_{n}}$$ (10)

$$\sigma_{UV}=\sum_{n=1}^{k}\left(\frac{\partial U}{\partial v_{n}}\right)\;\left(\frac{\partial V}{\partial v_{n}}\right)\;\;\sigma_{v_{n}}^{2}+\sum_{m\neq n}\left(\frac{\partial U}{\partial v_{m}}\right)\;\left(\frac{\partial V}{\partial v_{n}}\right)\;\;\sigma_{v_{m}v_{n}}$$ (11)

where *U* and *V* arc functions of (*v*_1_, …, *v_k_*), and the partial derivatives are understood to be evaluated at the average values of these variables. The variances of *v*_1_, …, *v_k_* are $\sigma_{v_{1}}^{2},\ldots ,\sigma_{v_{k}}^{2}$ and $\sigma_{v_{m}v_{n}}$ denotes the covariance of *v_n_, v_m_.*

As $\bar{x}$ is a function of 9 variables $\left(\bar{r},\bar{g},\bar{b},\bar{r_{1}},{\bar{g}}_{1},{\bar{b}}_{1},\bar{r_{2}},{\bar{g}}_{2},{\bar{b}}_{2}\right)$, $\bar{y}$ is a function of 6 variables $\left(\bar{r},\bar{g},\bar{b},L_{r},L_{g},L_{b}\right)$, and $\bar{z}$ is a function of 9 variables $\left(\bar{r},\bar{g},\bar{b},{\bar{r}}_{3},{\bar{g}}_{3},{\bar{b}}_{3},{\bar{r}}_{4},{\bar{g}}_{4},{\bar{b}}_{4}\right)$, the numbers of terms required by our problem can be counted readily. The numbers of terms in the variance equations are shown in table 1, and the numbers of terms in the covariance equations are shown in table 2.

Thus, for every wavelength there are required in a rigorous transformation from $\bar{r}\;\bar{g}\;\bar{b}$ to $\bar{x}\;\bar{y}\;\bar{z}$, 45 + 21 + 45 = 11 terms for the variances and 54 + 81 + 54 = 189 terms for the covariances, or a total of 300 terms for each wavelength. If the computation of the variances and covariances is performed at every 1 m*µ* step, as the $\bar{x}$, $\bar{y}$, $\bar{z}$ functions have been derived by Judd [6], from 360 to 830 m*μ* or at 471 wavelengths, some 141,300 terms would need to be computed.

Observe further that eqs (10) and (11) would include covariances between observations belonging to different spectral colors. That is, the possible correlation between (for example) ${\bar{g}}_{3}$ and ${\bar{g}}_{4}$ would be included in eq (10) and between ${\bar{b}}_{1}$ and ${\bar{b}}_{3}$ in eq (11).

## 5. Variances and Covariances in an Arbitrary Transformation

The foregoing procedure is completely general and rigorous for the conditions where empirical data are asked to indicate the choice of transformation constants, *k_i_*, for a transformation to a system analogous to the present CIE system. Where one wishes to make an arbitrary choice of transformation constants he indicates that the constants are without error or correlation with other variables.

In an arbitrary transformation the variances and covariances involving *k_i_* are chosen equal to zero thus:

$$V\left(k_{i}\right)=C\left(k_{i},k_{j}\right)=C\left(k_{i},v_{i}\right)=0$$ (12)

where *v_i_* denotes any of the variables, $\bar{r}$, $\bar{g}$, $\bar{b}$, *L*, with various subscripts. Thus in the transformation,

$$\begin{array}{c} {\bar{x}}_{\lambda }=k_{1}{\bar{r}}_{\lambda }+k_{2}{\bar{g}}_{\lambda }+k_{3}{\bar{b}}_{\lambda }\\ {\bar{y}}_{\lambda }=k_{4}{\bar{r}}_{\lambda }+k_{5}{\bar{g}}_{\lambda }+k_{6}{\bar{b}}_{\lambda }\\ {\bar{z}}_{\lambda }=k_{7}{\bar{r}}_{\lambda }+k_{8}{\bar{g}}_{\lambda }+k_{9}{\bar{b}}_{\lambda } \end{array}\}$$ (13)

the variance equations are:

$$\begin{array}{c} V\left(\bar{x}\right)=k_{1}^{2}V\left(\bar{r}\right)+k_{2}^{2}V\left(\bar{g}\right)+k_{3}^{2}V\left(\bar{b}\right)+2\left[k_{1}k_{2}C\left(\bar{r},\bar{g}\right)+k_{1}k_{3}C\left(\bar{r},\bar{b}\right)+k_{2}k_{3}C\left(\bar{g},\bar{b}\right)\right]\\ V\left(\bar{y}\right)=k_{4}^{2}V\left(\bar{r}\right)+k_{5}^{2}V\left(\bar{g}\right)+k_{6}^{2}V\left(\bar{b}\right)+2\left[k_{4}k_{5}C\left(\bar{r},\bar{g}\right)+k_{4}k_{6}C\left(\bar{r},\bar{b}\right)+k_{5}k_{6}C\left(\bar{g},\bar{b}\right)\right]\\ V\left(z\right)=k_{7}^{2}V\left(\bar{r}\right)+k_{8}^{2}V\left(g\right)+k_{9}^{2}V\left(\bar{b}\right)+2\left[k_{7}k_{8}C\left(\bar{r},\bar{g}\right)+k_{7}k_{9}C\left(\bar{r},\bar{b}\right)+k_{8}k_{9}C\left(\bar{g},\bar{b}\right)\right] \end{array}\},$$ (14)

$$\begin{array}{l} C\left(\bar{x},\bar{y}\right)=k_{1}k_{4}V\left(\bar{r}\right)+k_{2}k_{5}V\left(\bar{g}\right)+k_{3}k_{6}V\left(\bar{b}\right)+\left(k_{1}k_{5}+k_{2}k_{4}\right)C\left(\bar{r},\bar{g}\right)+\left(k_{1}k_{6}+k_{3}k_{4}\right)C\left(\bar{r},\bar{b}\right)+\left(k_{2}k_{6}+k_{3}k_{5}\right)C\left(\bar{g},\bar{b}\right)\\ C\left(\bar{x},\bar{z}\right)=k_{1}k_{7}V\left(\bar{r}\right)+k_{2}k_{8}V\left(\bar{g}\right)+k_{3}k_{9}V\left(\bar{b}\right)+\left(k_{1}k_{8}+k_{2}k_{7}\right)C\left(\bar{r},\bar{g}\right)+\left(k_{1}k_{9}+k_{3}k_{7}\right)C\left(\bar{r},\bar{b}\right)+\left(k_{2}k_{9}+k_{3}k_{8}\right)C\left(\bar{g},\bar{b}\right)\\ C\left(\bar{y},\bar{z}\right)=k_{4}k_{7}V\left(\bar{r}\right)+k_{5}k_{8}V\left(\bar{g}\right)+k_{6}k_{9}V\left(\bar{b}\right)+\left(k_{4}k_{8}+k_{5}k_{7}\right)C\left(\bar{r},\bar{g}\right)+\left(k_{4}k_{9}+k_{6}k_{7}\right)C\left(\bar{r},\bar{b}\right)+\left(k_{5}k_{9}+k_{6}k_{8}\right)C\left(\bar{g},\bar{b}\right) \end{array}\}.$$ (15)

To estimate these variances and covariances from the data obtained in color-matching by *n* observers, the following formulas are used:

$$\begin{array}{l} \hat{V}\left(\bar{r}\right)=\sum_{i=1}^{n}{\left({\bar{r}}_{i}-{\hat{\mu }}_{r}\right)}^{2}/\left(n-1\right)=\hat{\sigma }\frac{2}{r}\\ \hat{V}\left(\bar{g}\right)=\sum_{i=1}^{n}{\left({\bar{g}}_{i}-{\hat{\mu }}_{g}\right)}^{2}/\left(n-1\right)=\hat{\sigma }\frac{2}{g}\\ \hat{V}\left(\bar{b}\right)=\sum_{i=1}^{n}{\left({\bar{b}}_{i}-{\hat{\mu }}_{b}\right)}^{2}/\left(n-1\right)=\hat{\sigma }\frac{2}{b} \end{array}\}$$ (16)

$$\begin{array}{l} \hat{C}\left(\bar{r},\bar{g}\right)=\sum_{i=1}^{n}\left({\bar{r}}_{i}-{\hat{\mu }}_{r}\right)\left({\bar{g}}_{i}-{\hat{\mu }}_{g}\right)/\left(n-1\right)={\hat{\sigma }}_{\bar{r}\;\bar{g}}\\ \hat{C}\left(\bar{r},\bar{b}\right)=\sum_{i=1}^{n}\left({\bar{r}}_{i}-{\hat{\mu }}_{r}\right)\left({\bar{b}}_{i}-{\hat{\mu }}_{b}\right)/\left(n-1\right)={\hat{\sigma }}_{\bar{r}\;\bar{b}}\\ \hat{C}\left(\bar{g},\bar{b}\right)=\sum_{i=1}^{n}\left({\bar{g}}_{i}-{\hat{\mu }}_{g}\right)\left({\bar{b}}_{i}-{\hat{\mu }}_{b}\right)/\left(n-1\right)={\hat{\sigma }}_{\bar{g}\;\bar{b}} \end{array}\}$$ (17)

and

$${\hat{\mu }}_{r}=\sum_{i=1}^{n}{\bar{r}}_{i}/n,\;{\hat{\mu }}_{g}=\sum_{i=1}^{n}{\bar{g}}_{i}/n,\;{\hat{\mu }}_{b}=\sum_{i=1}^{n}{\bar{b}}_{i}/n.$$

The transformation equations derived by Kelly and Judd [6] and being considered for recommendation by the CIE for a 10°-field standard observer system for colorimetry are:

$$\begin{array}{l} {\bar{x}}_{10}=0.341080\;{\bar{r}}_{10}+0.189145\;{\bar{g}}_{10}+0.387529\;{\bar{b}}_{10}\\ {\bar{y}}_{10}=0.139058\;{\bar{r}}_{10}+0.837460\;{\bar{g}}_{10}+0.073316\;{\bar{b}}_{10}\\ {\bar{z}}_{10}=0.000000\;{\bar{r}}_{10}+0.039223\;{\bar{g}}_{10}+2.026200\;{\bar{b}}_{10} \end{array}\}.$$ (18)

As the constants, *k_i_*, of these equations were derived in a very complex way, the transformation will be treated as an arbitrary one.

## 6. Variability of Color-Mixture Data

The results of a statistical analysis of the Stiles-Burch color-matching data may be useful in determining the variances and covariances for the transformation to the proposed new CIE standard observer system. The Stiles-Burch data were analyzed because in the development of the new system they are being given greater weight than those of other investigators and because they are available in terms of individual observer data. Such an analysis was reported by Nimeroff, Rosenblatt, and Dannemiller [7] at the October 1959 meeting of the Optical Society of America and the results are given below.

The Stiles-Burch data were obtained under two experimental conditions. For condition I, 24 observers were used, while for condition II, 29 observers were employed. These two conditions differ in that the blue instrumental primary of condition I was located at 445.4 m*μ* (22,450 cm^−1^) while that of condition II was located at 470.6 m*μ* (21,250 cm^−1^). The data taken under these two conditions were transformed by Stiles-and Burch to an $\bar{r}\bar{g}\bar{b}$ system system in which the blue primary is located at 444.4 m*μ*. To test whether these two sets of transformed data are significantly different from each other the Hotelling *T*^2^ test [8] was used. The *T*^2^ test provides a comparison between the two sets of averages ${\bar{p}}_{1}$, representing ${\bar{r}}_{1}$, ${\bar{g}}_{1}$, ${\bar{b}}_{1}$, of condition I and ${\bar{p}}_{2}$, representing ${\bar{r}}_{2}$, ${\bar{g}}_{2}$, ${\bar{b}}_{2}$, of condition II in which the three correlated differences between corresponding averages are evaluated simultaneously against the variability present in the data from which the averages were estimated. In this test a high value of *T*^2^ implies that the differences between the averages compared are large. The *T*^2^ statistic in quadratic form for our problem of three variables in each of two conditions for *N*_1_ and *N*_2_ observers is:

$$T^{2}=\frac{N_{1}N_{2}}{N_{1}+N_{2}}\sum_{i,j=1}^{3}S^{ij}\left({\bar{p}}_{1i}-{\bar{p}}_{2i}\right)\left({\bar{p}}_{1j}-{\bar{p}}_{2j}\right),$$ (19)

where *S^ij^* is the (*i,j*) element of the inverse of the three by three matrix with elements

$$S_{ij}=\frac{1}{N_{1}+N_{2}-2}\left[\sum_{k=1}^{N_{1}}\left({\bar{p}}_{1ik}-{\bar{p}}_{1i}\right)\left({\bar{p}}_{1jk}-{\bar{p}}_{1j}\right)+\sum_{k=1}^{N_{2}}\left({\bar{p}}_{2ik}-{\bar{p}}_{2i}\right)\left({\bar{p}}_{2jk}-{\bar{p}}_{2j}\right)\right]$$ (20)

The subscripts 1 and 2 refer to the two conditions, *i* and *j* refer to the three primaries compared two at a time, and *k* identifies the observer for each condition.

**Figure 1:**
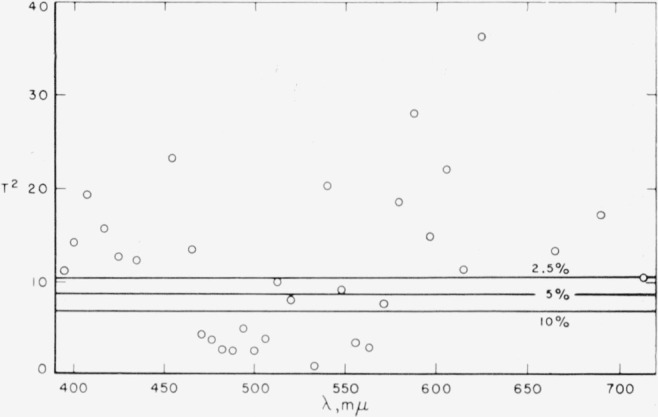
Values of the *T*^2^ statistic for evaluating differences between Stiles’ data obtained under condition I and under condition II. Critical values of *T*^2^ at three probability levels are indicated, showing that even at the 2.5% level the results obtained under the two conditions are for the most part significantly different.

The results of this test are plotted on figure 1 and indicate that the data obtained by Stiles under the two experimental conditions are in general statistically different even at the 2.5 percent level of significance, the exceptions lying between 470 and 580m*μ*. The difference may result from either an improper evaluation of the transformations of the two sets of data to a common primary system or an inherent dependence on the choice of instrumental primary. This latter supposition, if true, would invalidate any attempt to make the data obtained under the two sets of conditions comparable through such transformation. The differences between conditions I and II, while statistically significant, are in practical terms small.

**Figure 2:**
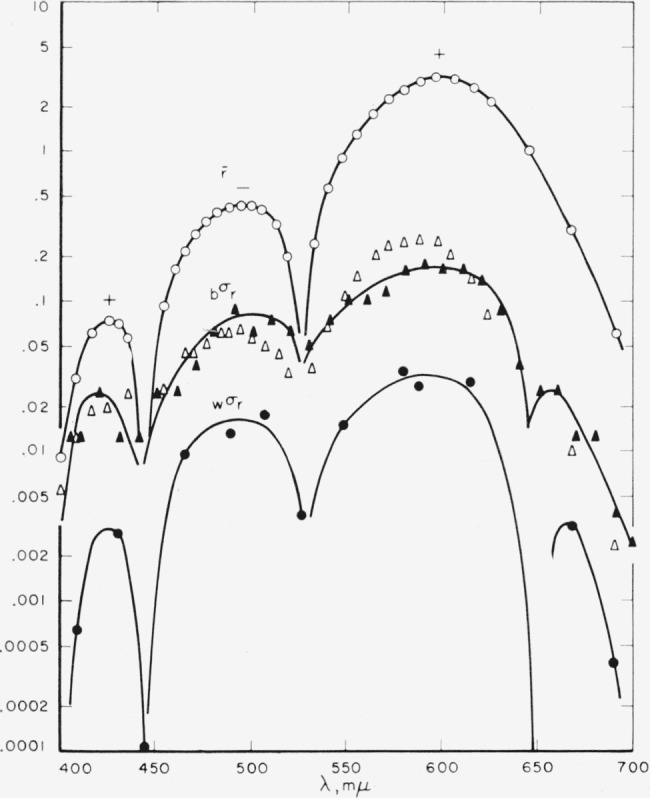
Means and variability of the red-primary data. Means, ○; standard deviations between observers (*_b_σ_r_*): Stiles Δ, Speranskaya ▲; standard deviations within observer (*_w_σ_r_*).

In spite of these differences the CIE is proceeding to average these data as well as those of Speranskaya for 27 observers, to derive the 10°-field tristimulus functions, $\bar{x}\bar{y}\bar{z}$. Figure 2 shows the averaged $\bar{r}$ function with its three lobes, two positive and one negative. Also shown are the estimates of between-observer standard deviations for the $\bar{r}$ function, based on the Stiles-Burch combined data and the Speranskaya data. Note that the standard deviations of these independent investigations are essentially of the same order of magnitude and range approximately from 10 to 20 percent of the mean function.

**Figure 3:**
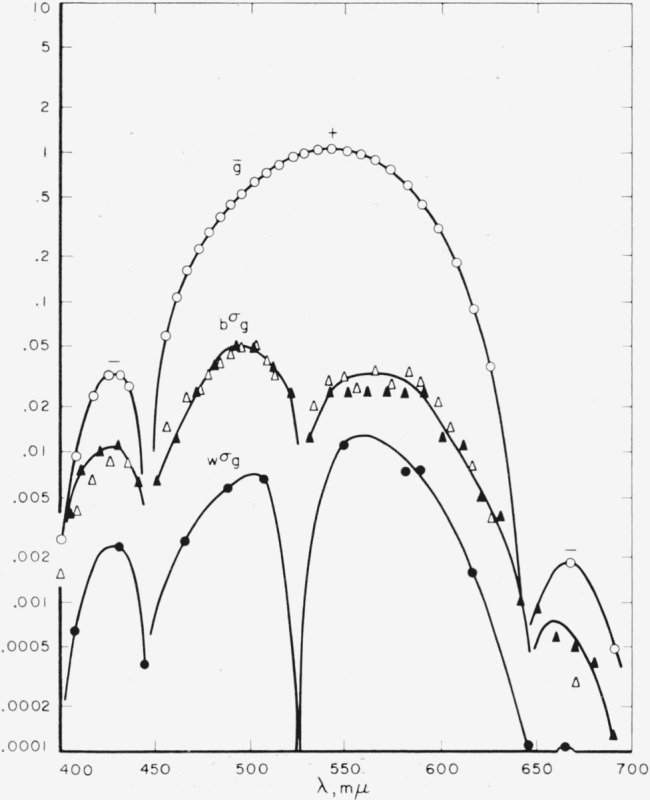
Means and varibility of the green-primary data. Means ○; standard deviations between observers (*_b_σ_g_*): Stiles Δ, Speranskaya ▲; standard deviations within observer (*_w_σ_g_*).

**Figure 4:**
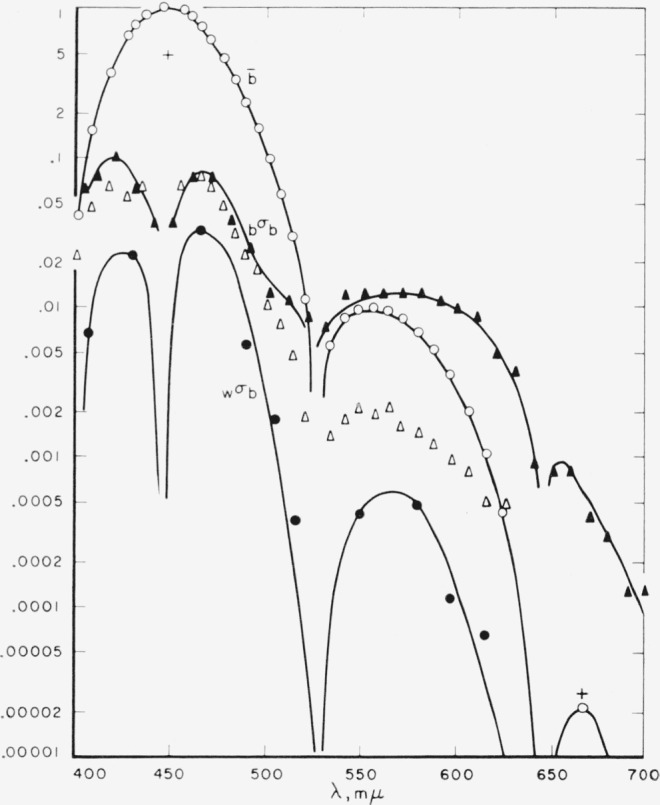
Means and variability of the blue-primary data. Means ○; standard deviation between observers (*_b_σ_b_*): Stiles Δ, Speranskaya ▲; standard deviation within observer (*_w_σ_b_*).

Strictly, the estimates shown in figure 2 (likewise figs. 3, 4) are estimates of the total standard deviation (see eq (3)). As the within-observer variances are relatively small, the estimated between-observer variances are only slightly over-stated. A satisfactory estimate of between-observer variance alone can be made only when the data include repeated observations by many (if not all) observers.

The lower curve gives the estimated within-observer standard deviations for the $\bar{r}$ function. These data are based on the average of individual variability of two observers, one repeating his measurements four times and the other repeating his measurements five times. As might be expected, the withm-observer standard deviations are approximately an order of magnitude lower than the between-observer standard deviations. Note that because settings in matches of the primaries themselves are assumed to be free from error the curves for the standard deviations are extended so as to approach zero at the location of the $\bar{r}\bar{g}\bar{b}$ primaries, that is at wavelengths 444.4, 526.3, and 645.2 m*μ*.

Figure 3 shows the same kind of information for the $\bar{g}$ function with its three lobes, two negative and one positive. Here also there is the same agreement between the Stiles-Burch data and the Speranskaya data; the within standard deviations are about an order of magnitude lower than the between standard deviations. The standard deviations approach zero at the location of the primaries.

Figure 4 shows the data for the $\bar{b}$ function. Here there is a disparity between the Stiles-Burch and the Speranskaya data in the longwave region. This disparity may result from the transformation from the instrumental primaries used in the Speranskaya investigation to those of the $\bar{r}\bar{g}\bar{b}$ system. It is more likely, however, that the procedure used by Stiles, that of employing for longwave matches a yellow primary at 588.2 rather than a green instrumental primary, is responsible lor the lower variability of the $\bar{b}$ function in this region.

**Figure 5:**
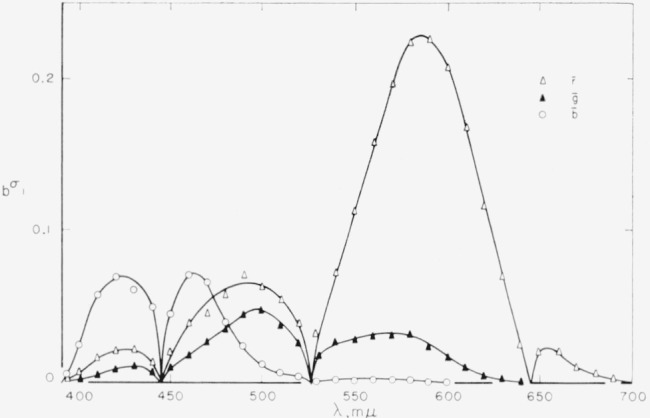
Mean standard deviation between observers for the Stiles-Burch and the Speranskaya data.

On figure 5 are shown averages of the between standard deviations derived from the data of the 53 Stiles-Burch observers and the data of the 27 Speranskaya observers. In the longwave (640 m*μ* and beyond) region of the $\bar{b}$ function the averages are based on the same weights used by Judd in deriving the mean $\bar{b}$ function itself from the British and Russian data. The weights for the British data are greater than those for the Russian data and increase with wavelength.

**Figure 6:**
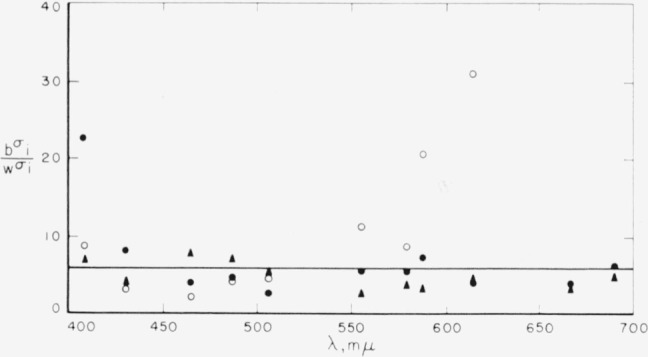
Ratio of the between to within standard deviations, for the three primaries, $\bar{r}\;\left(●\right)$, $\bar{g}\left(▲\right)$, and $\bar{b}\left(○\right)$, showing overall average ratio at 5.7.

It was noted above that the within standard deviations are about an order of magnitude lower than the between standard deviations. We are interested in learning whether the ratio of between to within standard deviations for the three functions can be represented by a single constant independent of wavelength and, if so, in determining the magnitude of such a constant. The computation of within-observer variability in a transformation to an $\bar{x}\bar{y}\bar{z}$ system would be simplified were this permissible. Figure 6 shows these ratios for the three color-mixture functions. The overall average ratio was derived from the geometric mean and is 5.7 in the spectral region 400 to 700 m*μ*. The geometric mean was used because of the wide disparity between the extreme values, namely 2.0 and 30.9. We consider this value reasonably representative of the ratio of between to within standard deviations, as better than 80 percent of the ratios he between half and twice 5.7.

In the transformations from color-mixture functions, $\bar{r}\bar{g}\bar{b}$, to tristimulus values, $\bar{x}\bar{y}\bar{z}$, covariances as well as variances need to be known. The correlation coefficient, *ρ_ih_*, relates the covariance, *σ_υ_*, to the variances, $\sigma_{i}^{2}$, $\sigma_{j}^{2}$ thus:

$$\rho_{ij}=\sigma_{ij}/\sigma_{i}\;\sigma_{j}.$$

**Figure 7:**
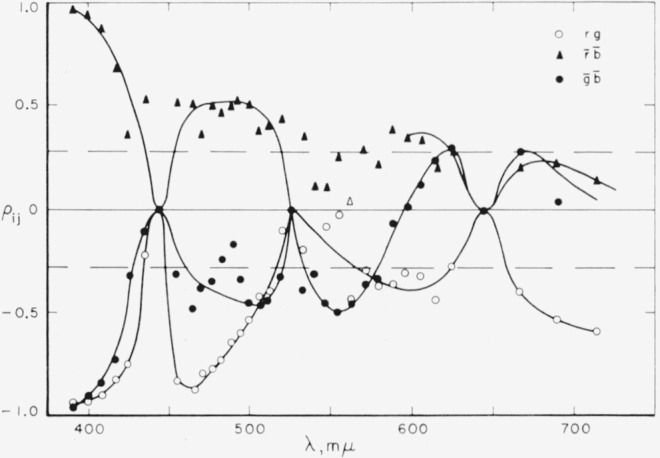
Correlation coefficients, ρ_ij_ for ij equal to $\bar{r}\bar{g}\;\left(○\right)$, $\bar{r}\bar{b}\;\left(▲\right)$, $\bar{g}\bar{b}\;\left(●\right)$. Dashed lines define the region within which these coefficients are not (statistically) significantly different from zero at the 5% level.

On figure 7 are plotted the correlation coefficients, $\rho \bar{r}\bar{g}\rho$, $\bar{r}\bar{b}$, and $\rho \bar{g}\bar{b}$. In these data, a positive correlation coefficient implies that if an observer uses more or less than average of one instrumental primary in making a match he uses more or less, respectively, than average of the other primary, also. Conversely, a negative correlation coefficient implies that if more or less than average of one primary is used, less or more, respectively, than average of the other primary is used. We note that $\rho \bar{r}\bar{b}$ is always positive or zero, while $\rho \bar{r}\bar{g}$ is almost always negative or zero. On the other hand, $\rho \bar{g}\bar{b}$ is negative in the shortwave spectral region, 380 to 590 m*μ*, and positive in the longwave region, 600 to 720 m*μ*.

Approximately two-thirds of the correlation coefficients are significantly different from zero at the 5 percent level of significance. The general consistency of sign and magnitude among values of the correlation coefficients at adjacent wavelengths provides further confirmation for the general pattern of values shown. Although some of the correlation coefficients are small in magnitude they should not be neglected in determining the variances and covariances in the $\bar{x}\bar{y}\bar{z}$ system transformed from the color-mixture data.

A few qualifications to the interpretation of the statistical analyses should be mentioned. First, statements concerning “statistical significance” are based on the assumption that the observations are normally distributed.

**Figure 8:**
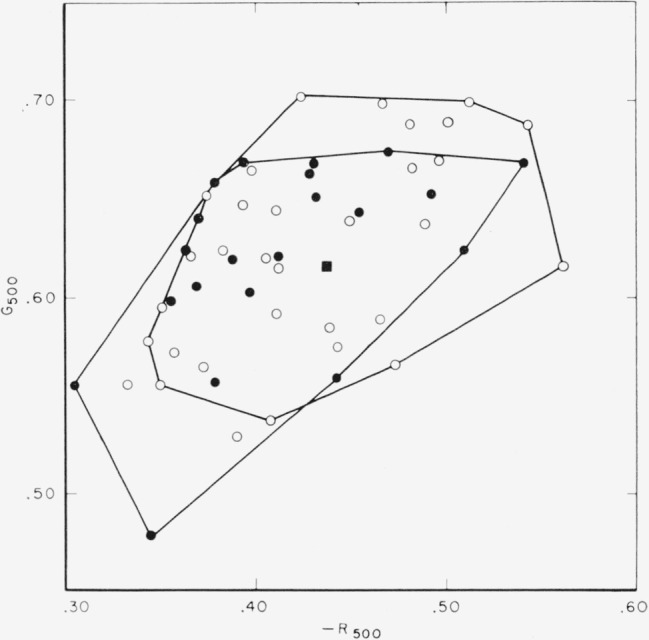
Plot of *R*_500_ against *G*_500_ for Stiles’ individual observers indicating no bimodality and very little skewness. Condition I (●), condition II (○). and mean (■). Straight lines join outlying values obtained under the two conditions.

There has been reported, however, by Judd [9] for 7 observers and by Wyszecki [10] for 10 observers, some evidence that the population of observers with normal color vision may be bimodally distributed. A limited analysis of the Stiles-Burch data for the 53 observers was undertaken to see if bimodality is revealed in their individual observer data. This analysis showed (see for example fig. 8 for *R*_500_ against *G*_500_) that although the distribution may be slightly skewed, there is insufficient data to conclude the existence of any bimodality. If more extensive data should lead to a reliable statement that the distribution of the population is bimodal, two standard observer systems might be required, one for each group.

A second qualification is required since possible correlations among observations for different spectral colors have been ignored because of the arbitrary choice of constants in the transformation. For example, the presence of correlation could account in part for the apparently consistent difference between conditions I and II, as shown in figure 1.

## 7. Complete Standard Observer System

A complete standard observer system should contain not only the mean spectral tristimulus functions, ${\bar{x}}_{\lambda }$, ${\bar{y}}_{\lambda }$, ${\bar{z}}_{\lambda }$, derived from the color mixture data, but should contain, also, the variances and covariances of these functions as derived from the within- and between-observer variability of the color mixture data.

Table 3 lists, for every 10 m*μ*, the spectral tristimulus values of the proposed standard observer system for colorimetry and the estimated total varianees, $V\left(\bar{x}\right)$, $V\left(\bar{y}\right)$, $V\left(\bar{z}\right)$, and covariances, $C\left(\bar{x},\bar{y}\right)$, $C\left(\bar{x},\bar{z}\right)$, $C\left(\bar{y},\bar{z}\right)$, for these spectral tristimulus values derived on the basis of an arbitrary transformation. It should be noted that the variances in table 3 are (5.7)^2^ or 32.5 times those in table 4.

## 8. Applications of the Complete System

As the individual observer data, on which the 1931 system is based, have been lost in antiquity, this system can be used only to determine what color matches the standard observer would make. A comprehensive analysis of color-mixture data in terms of means, variances, and covariances permits use of the proposed system to determine, in addition, the region of within and between uncertainties; that is, the extent to which a normal observer tends to make different matches on successive attempts, and the extent to which different normal observers vary one from another.

Some applications of the variances and covariances in the $\bar{x}\bar{y}\bar{z}$ system have been reported by Nimeroff [3] and by Wyszecki [11]. Further applications are sure to be suggested when a complete standard observer system for colorimetry, consisting of means, variances, and covariances, is established.

## Figures and Tables

**Table 1:** Number of terms in the variance equations

Variables	Variance terms	Covariance terms	Total
			
6	6	15	21
9	9	36	45

**Table 2:** Number of terms in the covariance equations

Variables		Terms
n	m	
		
9	6	54
9	9	81

**Table 3:** Proposed CIE tristimulus functions for 10°-field and between variances and covariances in $\bar{x}$, $\bar{y}$, $\bar{z}$

λ(m*μ*)	${\bar{x}}_{10}$	${\bar{y}}_{10}$	${\bar{z}}_{10}$	$V\left(\bar{x}\right)$	$V\left(\bar{y}\right)$	$V\left(\bar{z}\right)$	$C\left(\bar{x},\bar{y}\right)$	$C\left(\bar{x},\bar{z}\right)$	$C\left(\bar{y},\bar{z}\right)$
400	0.0191097	0.0020044	0.0860109	0.000126	0.00000118	0.00256	+0.0000104	+0.000568	+0.0000467
10	.084736	.008756	.389366	.000661	.0000110	.0132	+.0000708	+.00294	+.000321
20	.204492	.021391	.972542	.000937	.0000262	.0193	+.0000731	+.00421	+.000350
30	.314679	.038676	1.55348	.000737	.0000671	.0154	+.000106	+.00329	+.000509
40	.383734	.062077	1.96728	.000385	.0000448	.00978	+.0000816	+.00189	+.000355
450	.370702	.089456	1.99480	.000353	.0000561	.00815	+.0000413	+.00161	+.000185
60	.302273	.128201	1.74537	.00109	.0000995	.0206	−.0000151	+.00454	+.000136
70	.195618	.185190	1.31756	.00110	.000272	.0180	−.000101	+.00423	−.000131
80	.080507	.253589	0.772125	.000716	.000602	.00606	−.000108	+.00182	−.00000827
90	.016172	.339133	.415254	.000675	.00105	.00234	−.0000633	+.000973	+.0000759
500	.003816	.460777	.218502	.000414	.00129	.000593	+.000000553	+.000279	−.000245
10	.037465	.606741	.112044	.000325	.000829	.000190	+.0000884	+.000112	−.0000999
20	.117749	.761757	.060709	.000183	.000457	.0000649	+.000109	+.0000325	−.0000185
30	.236491	.875211	.030451	.000143	.000253	.0000271	+.000100	+.00000547	−.00000204
40	.376772	.961988	.013676	.000622	.000590	.0000431	+.000301	+.0000133	−.0000285
550	.529826	.991761	.003988	.00143	.000668	.000105	+.000528	+.0000531	−.0000700
60	.705224	.997340	.000000	.00283	.000847	.000100	+.000960	+ .000102	−.0000513
70	.878655	.955552		.00437	.000998	.0000942	+.00142	+.000147	−.0000107
80	1.01416	.868934		.00569	.00113	.0000778	+.00181	+.000184	+.0000381
90	1.11852	.777405		.00588	.000947	.0000652	+.00194	+.000194	+.0000806
600	1.12399	.658341		.00493	.000731	.0000379	+.00170	+.000148	+.0000720
10	1.03048	.527963		.00324	.000475	.0000248	+.00116	+.000102	+.0000518
20	0.856297	.398057		.00159	.000240	.0000123	+.000601	+.0000449	+.0000222
30	.647467	.283493		.000575	.0000918	.00000587	+.000226	+.0000120	+.00000591
40	.431567	.179828		.0000750	.0000127	.000000280	+.0000305	+.000000138	+.000000067
650	.268329	.107633		.0000470	.00000794	.000000050	+.0000191	+.000000052	+.000000032
60	.152568	.060281		.0000458	.00000713	.000000043	+.0000179	+.000000166	+.000000091
70	.0812606	.0318004		.0000129	.00000192	.000000016	+.00000492	+.000000084	+.000000044
80	.0408508	.0159051		.00000427	.000000619	.0000000017	+.00000161	+ .000000013	+.0000000077
90	.0199413	.0077488		.000000894	.000000128	.00000000043	+.000000335	+.0000000030	+.0000000016
700	.00957688	.00371774		.000000366	.000000052	.00000000016	+.000000137	+.00000000088	+.00000000049
10	.00455263	.00176847		.000000114	.000000016	.000000000039	+.000000042	+.00000000012	+.000000000086
20	.00217496	.00084619		.000000019	.0000000026	.0000000000043	+.0000000069	+.0000000000027	+.0000000000051

**Table 4:** Within variances in $\bar{x}$, $\bar{y}$, $\bar{z}$

λ (m*µ*)	$v\left(\bar{x}\right)$	$v\left(\bar{y}\right)$	$v\left(\bar{z}\right)$
400	0.00000390	0.0000000364	0.0000788
10	.000G204	.000000338	.000407
20	.0000288	.000000806	.000595
30	.0000227	.00000207	.000475
40	.0000119	.00000138	.000301
450	.0000109	.00000173	.000251
60	.0000337	.00000306	.000635
70	.0000340	.00000837	.000556
80	.0000220	.0000185	.000187
90	.0000208	.0000322	.0000720
500	.0000128	.0000396	.0000183
10	.0000100	.0000255	.00000587
20	.00000562	.0000141	.00000200
30	.00000440	.00000779	.000000834
40	.0000192	.0000182	.00000133
550	.0000441	.0000206	.00000324
60	.0000871	.0000261	.00000309
70	.000135	.0C00307	.00000290
80	.000172	.0000347	.00000240
90	.000181	.0000292	.00000201
600	.000152	.0000225	.00000117
10	.0000999	.0000146	.000000764
20	.0000489	.00000740	.000000380
30	.0000177	.00000283	.000000181
40	.00000231	.000000391	.00000000862
650	.00000145	.000000245	.00000000154
60	.00000141	.000000220	.00000000132
70	.000000398	.0000000591	.000000000493
80	.000000131	.0000000191	.0000000000524
90	.0000000275	.00000000394	.0000000000132
700	.0000000113	.00000000160	.00000000000493
10	.00000000351	.000000000493	.00000000000120
20	.000000000585	.0000000000801	.000000000000132

## Footnotes

1 Figures in brackets indicate the literature references at the end of this paper.
